# To Save Pangolins: A Nutritional Perspective

**DOI:** 10.3390/ani12223137

**Published:** 2022-11-14

**Authors:** Xin-Mei Wang, Geert P. J. Janssens, Chun-Gang Xie, Bo-Wen Xie, Zhi-Gang Xie, Hai-Jian He, Yan-Ni Wang, Jia Xu

**Affiliations:** 1 College of Chemistry and Life Sciences, Zhejiang Normal University, Jinhua 321004, China; 2 Key Lab of Wildlife Biotechnology, Conservation and Utilization of Zhejiang Province, Zhejiang Normal University, Jinhua 321004, China; 3 Department of Veterinary and Biosciences, Faculty of Veterinary Medicine, Ghent University, 9820 Merelbeke, Belgium; 4 Wildlife Protection and Management Station, Jinhua Municipal Bureau of Planning and Natural Resources, Jinhua 321052, China; 5 Department of Veterinary Medicine, Faculty of Agriculture, Jinhua Polytechnic, Jinhua 321000, China

**Keywords:** pangolins, natural diet, ex situ feeding, nutritive value

## Abstract

**Simple Summary:**

Pangolins are one of the world’s most trafficked mammals. Their numbers have decreased sharply due to their economic and assumed medicinal value in some parts of the world. Effective ex situ conservation requires appropriate nutrition to maintain a healthy population. However, due to the special feeding traits of pangolins and their high dependence on a natural ecosystem, many technical obstacles still limit the success of captive pangolin breeding. Therefore, based on the existing literature and practical experience, this review aims to compare the natural diet and successful diet of pangolins under human care, to outline the key factors of successful ex situ maintenance, and the strategies to improve their conservation success in animal care centers and in the wild.

**Abstract:**

Pangolins are one of the world’s most trafficked mammals. Since pangolins are highly adapted to ants and termites, they are important for controlling forest termite infestations. In addition to their ecological value, pangolins have economic and medicinal value. Currently, poaching and habitat destruction have radically reduced the number of pangolins, and *Manis pentadactyla*, *Manis javanica*, and *Manis culionensis* are now considered the most threatened pangolin species. In addition to the control of hunting and illegal trade, ex situ breeding is also a useful conservation method. However, many technical obstacles still limit the success of ex situ pangolin breeding. The special feeding traits of pangolins require a diet that meets nutritional and ethological needs. Based on the existing literature and practical experience, this review aims to compare the natural diet and successful diet in the human care of pangolins, to outline the key factors of successful ex situ maintenance from a dietary perspective, and the strategies to improve their conservation success in animal care centers and in the wild. The type of food used in successful pangolin protection agencies is quite variable in nutritional composition. In the diet of pangolins in the wild, the nutrient profile of different species of termites and ants and even the same species of termites and ants but different types (queens, soldiers, etc.) also displays differences. The crude protein content of some ants is higher than that of other foods, such as eggs, milk, and common cat food. The mineral and vitamin concentrations of ants also exceed many common food items, such as oil, meat, and eggs. However, not much is known about the bioavailability of minerals from ants and termites. Based on comparisons between foods, it is clear that the main difference between diets in the wild and in human care of pangolins is that the latter contains fewer insects and vitamins, such as vitamin E, vitamin A, and vitamin B2, and more carbohydrates and non-protein substances than the former. Although many successful dietary formulae have been developed, the pangolin’s nutritional needs are still less well studied. A diet with the nutrient concentrations observed in the wild may add to successful ex situ conservation.

## 1. Introduction

Pangolins are myrmecophagous mammals of the genus *Manis*, order Pholidota, class Manidae. Currently, there are eight species: four in Asia and four in Africa [1]. The pangolin has no teeth in adulthood and mainly captures ants and termites with a long tongue. It is estimated that an adult pangolin can eat more than 70 million insects a year and has a significant impact on forest termite population control [2]. The assumed medicinal and gastronomic value of pangolins brings great economic benefits for illegal traders, which leads to a dramatic decline in their wild populations [3]. According to the former State Forestry Administration of the People’s Republic of China, the number of wild pangolins declined from 64,000 to 25,100–49,450 from 1998 to 2008 [4]. In order to further strengthen the protection of pangolins, the Chinese government upgraded all pangolin species from national Grade II protected wildlife to national Grade I protected wildlife on 5 June 2020 [5]. Later, different from the 2015 edition, the 2020 edition of the Chinese Pharmacopoeia removed the pangolin, and it is no longer included as traditional Chinese medicine [6].

Although the change in these policies can better protect pangolins and their habitat, ex situ breeding is a more effective way to protect endangered pangolins. However, the transition of pangolins from wild ants to a “gruel” diet under human care is difficult, and the pangolin’s nutritional needs are less well studied, which is why providing adequate nutrition is the biggest barrier to ex situ breeding [7]. Studies have suggested that pangolin health is related to nutrition and parasite infection. For example, some pangolins suffer from gastrointestinal diseases and die of malnutrition because the artificial food provided is not suitable for their digestive system [8,9]. There have also been intestinal parasites and nutritional failures caused by prolonged starvation [10]. Even pangolins that are able to eat artificial food on their own also die of gastric perforation disease due to food inadaptability [11]. Therefore, it has been postulated that malnutrition is the main reason pangolins cannot thrive under human care. That preference may vary with environmental conditions, and the ability of pangolins to adapt to different foods is also different. Pangolins’ food in the wild and food under human care are quite different in type and nutrient composition [12]. It is important to pay more attention to the diet of pangolins under human care and confiscated, rescued wild pangolins [13]. This review focused on the comparison of food resources in the wild and ex situ food resources to identify nutrients that may need more consideration in ex situ breeding of pangolins. 

## 2. The Diet of Pangolins in the Wild

### 2.1. The Type of Food in the Wild

The nutritional needs of many wildlife species are still largely unknown and how they are fed is often based on a “model” species for which there is already an understanding of their nutritional needs [12]. The giant anteater (*Myrmecophaga tridactyla*) serves as a “model” species of the pangolin because it has a similar feeding ecology [14]. The food of pangolins in the wild consists mainly of termites and ants. Field studies have shown that in addition to ants and termites, a pangolin’s diet includes insect larvae, bees (pupae), flies, earthworms, crickets, and some other arthropods, sometimes also sand, small grains, and grass during feeding [15,16]. The study also found that the diet composition of pangolins changed with latitude and season. For example, in summer, it is easy to find ants on the ground, while termites hide in underground tunnels, so ants are the main source of food for pangolins; in winter, when ants move into underground nests because of low temperature, pangolins prefer termite nests because their biomass is larger than that of ants [15].

Although the natural pangolin diet is quite specialized, they might not have a particular preference for ant species. Swart et al. [17] found that *Smutsia temminckii* feeds on 15 kinds of ants and 5 kinds of termites without an obvious preference. The literature reported on 18 kinds of food records of *Manis pentadactyla* in the wild; these included nine species of termites and nine species of ants [9]. However, a study found that some ants were unacceptable to pangolins, such as *Manis pentadactyla* in the Fog Ridge Reserve, which disliked *Paratrechina bourbonica* and *Odontotermes zunyiensis* [16,18]. Therefore, the researchers speculated that the nutritional composition, palatability, and safety of food are important factors in pangolin food selection.

### 2.2. Nutrient Composition of the Pangolin Diet in the Wild

The nutrient profile of different ants and even ants of the same species can display differences. For example, the protein contents of *Polyrhachis vicina* Roger male, female, and *Polyrhachis lamellidens* Smith were 64.50%, 64.10%, and 58.60%, respectively; the fat contents were 9.50%, 8.57%, and 8.52%, respectively; and Zn, Ca, Mg, and Fe contents were 138–155, 307–613, 164–208, and 378–413 mg/kg, respectively [19]. Similar differences occurred between queens, soldiers, and workers of *Macrotermes bellicosus*, where crude protein concentration in the soldiers was higher than in workers and queen termites; worker termites had the highest vitamin C content, while queens had the highest vitamin A content [20]. The metadata of the animals are shown in Table 1 and Supplementary Table S1. Therefore, appropriate feeding of pangolins will likely need to go beyond providing any random source of ants or termites. Ants are the representative food of pangolins in the wild, and the ratio of non-protein energy to protein energy (NPE/PE) of ants is plotted using the nutritional geometric framework [21]. Currently, the closest approximation of NPE/PE ranges between 0.48:1 and 1.2:1 [12]. However, nutrition selection involves much more than just a ratio: this approach only allows us to visually compare the major macronutrients in the diet.

Table 1 shows the variation in macronutrient concentrations among a selection of ants and termites. In general, it shows a diet that is high in crude protein, with varying levels of fat, minor amounts of carbohydrates, and varying levels of ash (hence minerals). A particular comment on the reported crude protein concentrations is that crude protein determination does not discern real protein from non-protein nitrogen fractions. In the case of insects with a large exoskeleton proportion, chitin can make up a considerable part of the crude protein fraction. Therefore, crude protein concentrations in ants and termites should not be taken as an indicator of real protein requirements in pangolins. Depending on whether pangolins express endogenous chitinase in their digestive tract, chitin will either be a digestible energy source or a substrate for microbial hindgut fermentation. According to the report, the high expression of an acidic mammalian chitinase, produced mainly in the oxyntic glands of the stomach of *Manis javanica*, indicated that the gastrointestinal tracts of *Manis javanica* had evolved an enzymatic adaptation, turning at least part of the chitin into a digestible energy source rather than only a substrate for microbial hindgut fermentation [26]. Nevertheless, it will not provide amino acids to build protein but will rather add to the energy sources. The varying ash content is likely to reflect the lifestyle of the insects, i.e., some species will eat soil, hence loading their guts with ash content [27]. 

**Figure 1:**
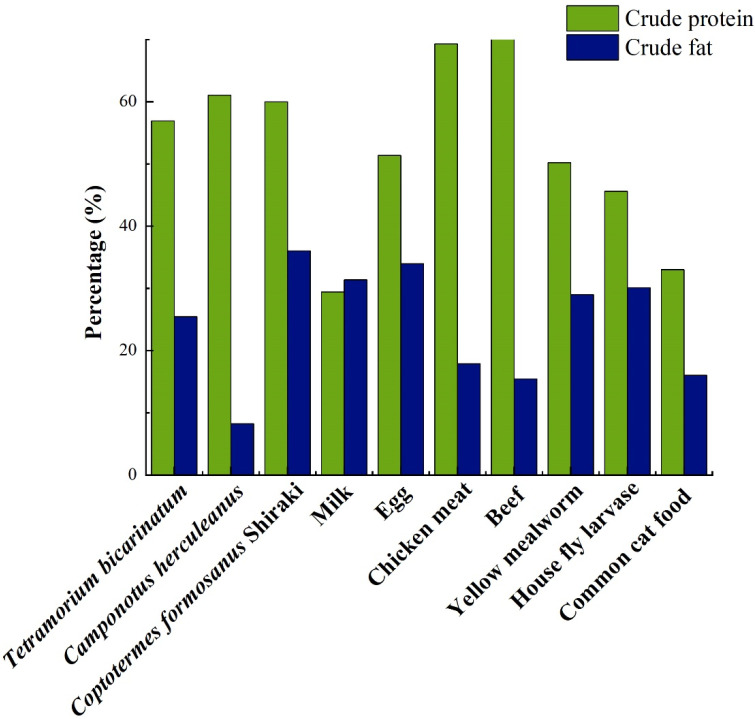
Macronutrient concentrations in natural and other feeds of pangolins (dry weight) [22,30,31]. Notes: The other feeds include milk, egg, chicken meat, beef, yellow mealworm, house fly larvase and common cat food.

As shown in Figure 1, the crude protein content of pangolin’s natural foods, such as *Tetramorium bicarinatum*, *Camponotus herculeanus*, and *Coptotermes formosanus*, was significantly higher than that of other foods, such as eggs, chicken, and common cat food. In addition, the other nutrient concentrations of ant insects and their fungus beds, such as *Formica rufa* L. and *Odontotermes formosanus* Shiraki, were also very high (Supplementary Tables S2–S4). In addition to basic nutrients, ants were also rich in formic acid, which is mainly used for defense, trace marking, and antibacterial action. For example, wood ants produced large quantities of formic acid in their venom gland, which they readily sprayed to defend or disinfect their nest [28]. A recent study on a semi-free-ranging group of capuchin monkeys (*Cebus apella* L.) found that they rub ants or other arthropods on themselves, releasing volatile substances in their fur to repel ectoparasites such as ticks [29]. 

## 3. The Food for Pangolins under Human Care 

### 3.1. Diet Resources under Human Care

The requirement of ant breeding is strict on temperature and humidity, and the breeding time is from March to October, so the availability of ants is reduced at other times [32,33,34]. For larger-scale care of pangolins, there are not enough natural ant resources to meet their nutritional needs, which implies insufficient food supply for pangolins under human care and will affect the display of natural behavior [35]. The development of a suitable artificial pangolin food is thus necessary for successful ex situ conservation. 

Based on the natural food ingredients of pangolins in the wild, many recipes have been developed for pangolin husbandry, including eggs, meat (ground beef, horse meat, fish), milk, milk powder, orchid leaves, carrots, yeast, multivitamins, and insects [36]. Taipei Zoo has been very successful in raising and breeding pangolins, which is largely related to the gradual development of a suitable diet. Their pangolin diet formula was developed from 1989 to 1995; the researchers optimized a diet for pangolins, consisting of 100 g of mixed silkworm powder (silkworm powder, yeast powder, coconut powder, ratio of 10:2:1), 100 g of bee larvae, 50 g of mealworms, 1 egg yolk, a quarter of an apple, and 0.5 mL infant multivitamin solution [7]. Pangolins are adaptable to these artificial diets. Cheng et al. (2000) reported on an artificial diet containing milk, porridge, *Crematogaster rogenhoferi* Mayr, *Polyrhachis lamellidens* Smith, locust leaf powder, a multivitamin mixture, glucose, and egg yolk, which was able to keep pangolins healthy [13]. Lu et al. (2014) produced five feed formulas, which included 45% fresh milk, 45% ant powder, and 10% minerals and vitamin supplements and found that pangolins gained weight, and daily feed intake was higher than in other dietary combinations, such as the combination of 30% milk powder, 20% cooked egg yolk, 40% ant powder, 10% supplements, and 30% earthworm powder; 20% cooked egg yolk, 40% ant powder, and 10% supplements; etc. [37]. Gao et al. (2017) used corn flour, mealworm (*Tenebrio molitor*), soybean powder, fish meal, ant powder, and some food additives (multiple vitamin tablets, salt, glucose) to feed *Manis javanica* and found that pangolins had a normal body condition without weight change [38]. Overall, research on the captivity diet of the pangolins has increased their food intake compared with before and displayed gradually improved health and survival rates.

### 3.2. Nutritional Composition Analysis of Food under Human Care

Due to differences in geographical environment and biological abundance, the types of food and nutritional composition in different pangolin protection agencies show great differences. For example, in Wildlife Reserves Singapore (WRS-Singapore), Ragunan Zoo (Indonesia), Save Vietnam’s Wildlife (SVW-Vietnam), Taipei Zoo (Taiwan), Ueno Zoo (Japan), Leipzig Zoo (Germany), Nandankanan Zoo (India), and Chongqing Normal University (China), diets range in their levels of invertebrates (ants, red weaver ants, green weaver ants, weaver ant eggs, red ants, bee larvae, silk worms, and/or mealworms), vertebrates (beef meat, eggs, and/or egg yolks), plant matter (coconut husk, apples, corn flour, and/or soya beans), concentrates (cat kibbles, hedgehog pellets, and/or insectivore pellets), dairy (yoghurt), and supplements (clay, chitin powder, calcium lactate, vitamin B, vitamin A, vitamin K, choline chloride, and/or olive oil) and water [12]. As shown in Table 2, there was a high variability in the nutrient content of diets across institutions: crude fat ranged from 18.56% to 31.27%, crude protein from 32.41% to 55.11%, acid detergent fiber (ADF) from 4.61% to 16.01%, neutral detergent fiber (NDF) from 9.12% to 18.94%, Ca 0.15% to 1.27%, P from 0.23% to 0.84%, and water soluble carbohydrates (WSCs) from 1.60% to 25.84% [12]. Nutrient concentrations of pangolin artificial foods such as eggs, milk, chicken, and beef are different on a both dry and fresh matter basis. The specific values are shown in Table 3.

## 4. Comparison between Diets in the Wild and Diets under Human Care

For most pangolins that move from the wild to captivity, it is important to provide a diet that allows a smooth adaptation to the new environment. The diet under human care contains fewer insects, with carbohydrates, fat, and non-protein substances much higher than the diet in the wild [12]. At the same time, due to availability issues, the proportion of ants in the ex situ diet for pangolins is much lower than that for wild pangolins, but pangolins under human care can receive more abundant and stable food, which offers more certainty regarding nutrient intakes for improving the survival of pangolins under human care [25,40], but at the same time, it increases the responsibility to get the diet composition right. In addition, the intake and energy of artificial food in pangolins is easier to determine than that of wild pangolins. For example, the gross energy (GE) of the three artificial food formulas formulated by Taipei Zoo were 0.59 MJ, 0.45 MJ, and 0.44 MJ, respectively [39].

**Figure 2:**
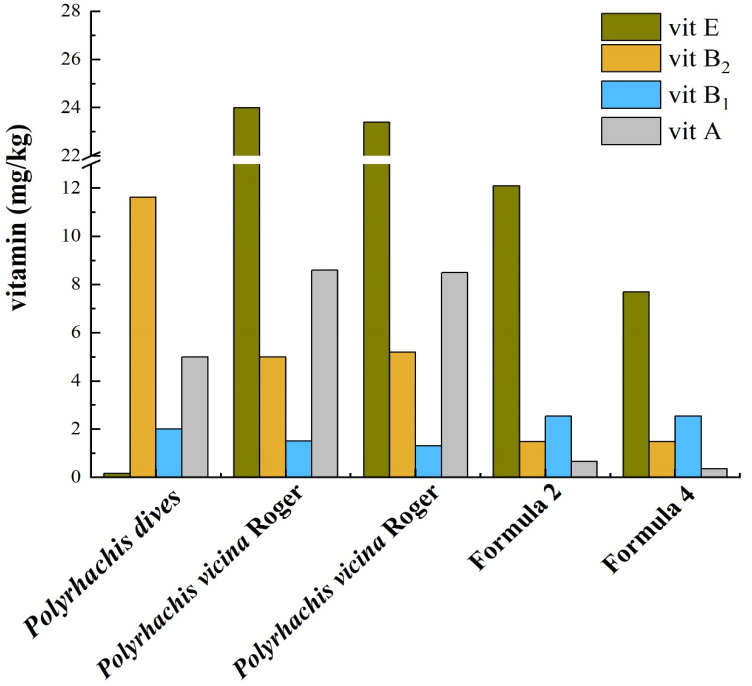
Vitamin concentrations in natural food and formula under human care of pangolins [46,47,48,49]. Notes: Formula 2 includes cat food, ant powder, yellow mealworms, eggs, apples, water, amino acid, astragalus, and trace elements; formula 4 includes cat food, ant powder, yellow mealworms, eggs, apples, carrots, cicada pupas, water, and amino acid. The concentration of vitamin B1 is less than 2 mg/kg, and the concentration of vitamin A is less than 5 mg/kg in *Polyrhachis dives*.

**Figure 3:**
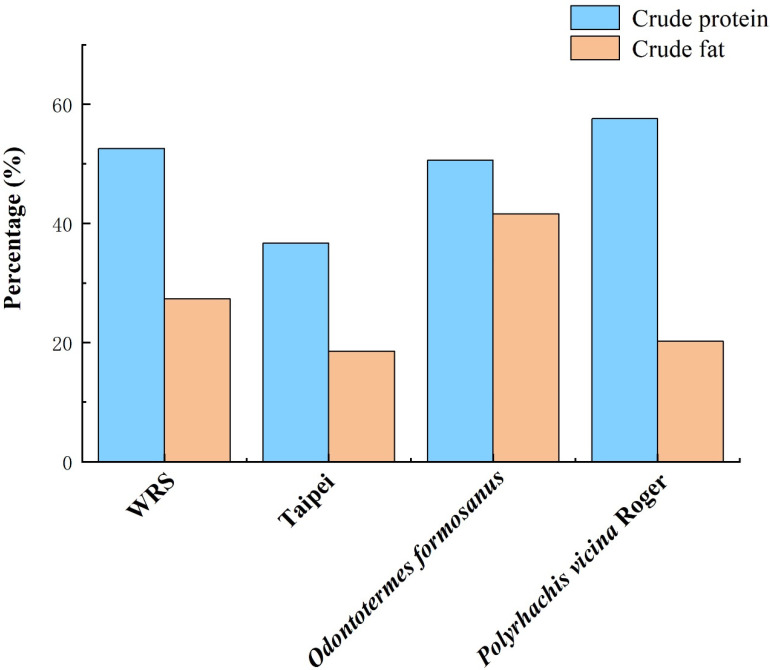
Macronutrient concentrations in natural and artificial foods of pangolins (dry weight) [8,12,23]. Notes: WRS, Wildlife Reserves Singapore (Singapore); Taipei, Taipei Zoo (Taiwan).

Liu et al. (2021) compared the artificial diets of Guangzhou Zoo and Shenzhen Wildlife Rescue Center with published wild ant nutrient profiles and found that the WSC and NDF of wild ants were lower, but the protein and fat contents were higher, with a similar ADF level [12,40]. Yang et al. (1996) analyzed the nutrients of *Polyrhachis vicina* Roger, *Oecophylla smaragdina* Fabricius, and *Macrotermes denticulatus* and found that these three insects not only had a high protein content (between 49.92% and 61.52%), but also mineral and vitamin concentrations that exceeded those of many common food items, such as oil, meat, eggs, and fish [25]. The fact that mineral concentrations in ants are often distinctly higher than those in common muscle meat (pork, chicken, beef, fish, shrimp) resources is confirmed by other authors [30,44]. Not much is known about the bioavailability of minerals from ants and termites, but the observation of these high mineral concentrations in typical diet items for pangolins warrants investigations into the mineral requirements and metabolism of pangolins (Supplementary Table S5). Micronutrient requirements are often overlooked when feeding wild animals under human care, whereas there are plenty of examples in the literature regarding cases of health issues associated with potential micronutrient malnutrition. For example, Zhang et al. (2019) observed a lack of B vitamins in the artificial diets of pangolins, which led to the occurrence of vitamin B deficiency diseases with clinical signs of skin inflammation [45]. In Figure 2, there are obvious differences in vitamin concentrations between diets of pangolins in the wild and diets of pangolins under human care. For example, the artificial foods of pangolins had significantly lower amounts of vitamin A than foods consumed in the wild. As shown in Figure 3, the natural and artificial food of pangolins also had the same differences in crude protein and crude fat.

## 5. Other Factors Affecting the Nutrition of Pangolins

### 5.1. Digestibility of Food

A previous dissection of a dead pangolin found an obvious sphincter in its pylorus, which controlled the speed of food leaving the stomach to ensure that the food was fully ground and mixed with gastric juice [50]. Imai et al. (1973) found that the pangolin’s stomach had developed muscles, which sometimes contained small stones and gravel that likely help with digestion [50]. One study found that their weight doubled after 15 months of continuous feeding with *Manis pentadactyla*, indicating that their metabolism is slow, and overfeeding should be avoided to reduce excessive growth or adult obesity [39]. Important in evaluating digestibility is the fact that the main items in the natural diet (ants and termites) have exoskeletons with high levels of chitin [46,50]. Chitin is the most abundant biopolymer in nature after cellulose. It exists, for instance, in the exoskeleton of insects and crustaceans, the endoskeleton of mollusks, and the cell walls of fungi. It is difficult to digest in most digestive systems, although many insectivorous species show secretion of endogenous chitinases in their digestive tract [51]. Researchers at Taipei Zoo have found that chitin may increase the species’ apparent digestibility of organic matter [12]. Adding 5% ground chitin can improve the fecal consistency of *Manis pentadactyla.* It is speculated that adding chitin to the diet may be a practical method to control the weight of pangolins in captivity [39]. Cabana found that the addition of food containing more chitin to the pangolin’s diet may increase the average retention time of food in the pangolin stomach [52]. Therefore, adding chitin in the artificial diet might be beneficial for the gut health of pangolins partially due to the impact on digestibility. It has been reported that the addition of peat to the diet could also improve the fecal consistency of the small anteater [53,54,55]. Moreover, a study reported that adding plantain seed powder to pangolins’ diet improved their fecal quality, suggesting that adding plantain seed powder may help in maintaining gastrointestinal function in this species [55].

### 5.2. Diet Reflected in Feces

Fecal samples can be very useful for studying the diet and habitat of the animals. DNA metabarcoding using high-throughput sequencing technology can also be used for diet analysis with high sensitivity and greatly reducing the cost and time of analysis [56,57]. For example, in 1997, Reed used PCR technology to study the species and sex of seal food to learn about competition for food between seals and carnivorous fish [53]. At the same time, stable isotope analysis can also be used to study diet analysis for animals [54]. For now, only fecal microscopic analysis data are available. A large number of ant and termite epidermises were found in pangolin feces, including their heads, legs, and abdominal epidermis, thus allowing an estimation of pangolins’ prey frequency and prey composition [18,58]. However, the exoskeletons of termites and ants are different in physical structure. Termites have a thin and pliable epidermis, reduced forefeet, and slender legs, compared to the harder epidermis and exoskeletons of ants [18,59]. Therefore, the digestibility of termites and ants for pangolins may vary depending on their exoskeleton’s hardness and thickness. Recovery in pangolins feces between ants and termites might be different, but there is no evidence to support this hypothesis.

### 5.3. Intestinal Microbiota

Digestive enzymes and gut microbes play an important role in animal digestion, the gut microbiota influences the host metabolism, nutritional balance, and the immune response, but there are few reports regarding the pangolin’s digestive enzymes and intestinal microbes [60,61]. The intestinal microbial community of *Manis javanica* is similar to that of herbivores, but different from that of anteaters and other myrmecophagous animals [61]. Different diet, environment, and captivity time can change the gut community composition and abundance of pangolins. Liu et al. (2021) compared ex situ and wild pangolins’ gut microbes and found that the intestinal communities of short-term ex situ pangolin populations were very similar to those of the wild populations, whereas those of long-term ex situ pangolin populations were quite different [40]. Despite their different diet and living environment, we still find that the gut of pangolins contains the family Clostridiaceae, which is highly abundant in carnivores, such as cheetahs [62], wolves [63], dogs [63], and the genera *Clostridium* and *Cellulosilyticum*. The genera *Clostridium* and *Cellulosilyticum* were all involved in the dietary fiber metabolism [64]. However, the difference was that the abundance of these organisms in captivity was significantly higher than in the wild in terms of family and genus levels by linear discriminant analysis effect size (LEfSe) [40]. Thus, the fiber-metabolizing microbiota might be enhanced through the high plant fiber content in artificial diets. Both also showed varying degrees of enrichment in terms of some metabolic pathways. For example, pangolins under human care exhibited an increased capacity for carbohydrate and fatty acid metabolism and short-chain fatty acid synthesis, but reduced ability to metabolize exogenous substances [40]. This may be directly related to the fact that they live in different environments and are exposed to different substances. Previous studies have found that many species of *Clostridium* are pathogens that are associated with gastrointestinal diseases, and a bacterium carrying the virulence factor GroEL was also more abundant in pangolins under human care [40]. This may also be the main reason pangolins under human care are more susceptible than wild pangolins. We must, however, add that Clostridiaceae are a normal dominant group in the gut microbiome of wild carnivores [36,65].

To understand the digestive function of pangolins, Zhang et al. [66] analyzed the protein components in the salivary and intestinal fluids of *Manis javanica*. There was a significant difference in protein types between the two digestive fluids. There were 727 different protein types in saliva and 2968 different protein types in intestinal fluid. In digestive enzyme expression analysis, there were five pathways in intestinal fluid related to carbohydrate digestion and absorption, protein digestion and absorption, fat digestion and absorption, vitamin digestion and absorption, and mineral digestion and absorption. Different from intestinal fluid, there was no route of vitamin digestion and absorption in the saliva [66]. Similarly, researchers have found that the gut microbiota of ex situ and wild groups were distinct with respect to certain metabolic pathways. For example, the abundance of xenobiotics’ biodegradation and metabolism and terpenoids and polykeones in the wild *Manis javanica* was significantly higher than that in the short-term ex situ pangolin groups [40]. However, the abundance of pathways related to fatty acid metabolism, such as arachidonic acid metabolism, linoleic acid metabolism, and alpha-linolenic acid metabolism, were significantly higher in the *Manis javanica* captivity group than the wild group [40]. Therefore, ex situ and wild pangolins exhibited distinct differences in the composition and functionality of the gut microbiota. 

## 6. Conclusions and Perspective

Ex situ breeding is an important strategy to protect pangolins, with nutrition being one of the most critical factors. While many successful dietary formulae have been developed, a thorough nutrient analysis of the items in the pangolin diet in the wild is required. The ant epidermis contains chitin, and the body contains formic acid, formaldehyde, and other substances, which may support digestive characteristics and resistance against various pathogens. The present work points to specific nutrient patterns (amino acids, fatty acids, minerals, vitamins) in the natural pangolin diet that may have an importance in successful ex situ conservation. Insights in the selection, digestibility, and metabolization of chitin and these other nutrients in pangolins is still lacking and may be needed to support successful conservation of pangolins. 

## Figures and Tables

**Table 1:** Macronutrient concentrations across ant and termite species (g/100g).

Species	Crude Protein	Crude Fat	Carbohydrate	Moisture	Crude Ash	References
*Tetramorium bicarinatum*	56.91	25.44	8.42	/	/	[22]
*Camponotus herculeanus*	61.03	8.25	15.78	/	/	[22]
*Coptotermes formosanus*	50.62	41.60	0.48	/	/	[22]
*Odontotermes formosanus*	50.62	41.60	0.42	/	/	[8]
*Myrmica rubra*	56.81	25.45	7.90	/	/	[8]
*Polyrhachis dives*	56.15	25.10	7.20	/	/	[8]
*Polyrhachis vicina* Roger	57.60	20.20	/	7.10	11.10	[23]
*Formica truncicola* Forel	56.60	29.10	3.50	7.30	3.50	[24]
*Oecophylla smaragdina*	57.89	16.62	10.46	8.94	4.19	[25]
*Macrotermes denticulatus*	49.92	14.21	9.89	11.20	3.60	[25]

/ = The data were not measured.

**Table 2:** Nutrient concentrations of each institution’s diets on a dry matter basis.

Institution	Crude Protein	Crude Fat	ADF	NDF	Ca	P	WSC	References
	%	%	%	%	%	%	%	
WRS	52.58	27.33	10.17	12.09	0.22	0.25	1.58	[12]
Ragunan	50.86	24.63	9.76	15.80	0.15	0.83	3.06	[12]
SVW	53.68	31.27	8.86	9.12	0.25	0.67	1.60	[12]
Taipei 1	36.70	18.56	14.63	15.49	0.84	0.84	24.19	[12]
Taipei 2	40.00	/	/	/	2.50	0.50	/	[39]
Taipei 3	32.20	/	/	/	2.00	0.40	/	[39]
Shenzhen	33.18	3.45	13.06	25	0.21	0.34	2.11	[40]
Guangzhou	47.22	14.23	9.17	23.33	1.73	0.76	19.84	[40]
Ueno	32.41	27.51	4.61	9.44	0.94	0.67	25.84	[12]
Leipzig	32.69	23.82	9.14	15.34	1.27	0.37	21.59	[12]
Nandakannan	55.11	20.13	/	/	/	/	/	[41]
Chongqing	37.11	28.65	16.01	18.94	/	/	12.29	[42]

Notes: WRS, Wildlife Reserves Singapore (Singapore); Ragunan, Ragunan Zoo (Indonesia); SVW, Save Vietnam’s Wildlife (Vietnam); Taipei, Taipei Zoo (Taiwan); Shenzhen, Shenzhen Wildlife Rescue Center (China); Guangzhou, Guangzhou Zoo (China); Ueno, Ueno Zoo (Japan); Leipzig, Leipzig Zoo (Germany); Nandankanan, Nandankanan Zoo (India); Chongqing, Chongqing Normal University (China). ADF, acid detergent fiber; NDF, neutral detergent fiber; Ca, calcium; P, phosphorus; WSC, water soluble carbohydrate. / = The data were not measured.

**Table 3:** Comparison of some items in the diet of zoo pangolins based on a dry and fresh matter basis.

Species	Crude Protein (%)	Crude Fat (%)	References
Milk (dry matter basis)	29.41	31.37	[43]
Egg (dry matter basis)	51.35	33.98	[43]
Chicken meat (dry matter basis)	69.29	17.86	[43]
Beef (dry matter basis)	73.16	15.44	[43]
Egg (fresh matter basis)	13.30	9.40	[22]
Milk (fresh matter basis)	3.00	2.90	[22]
Chicken meat (fresh matter basis)	18.50	11.20	[30]
Beef (fresh matter basis)	19.90	13.10	[30]

## Data Availability

Not applicable.

## Supplementary Materials

The following supporting information can be downloaded at: https://www.mdpi.com/article/10.3390/ani12223137/s1. Table S1: Macronutrient concentrations across ant and termite species (g/100g) [8,22,23,24,25,30,67]; Table S2: Fatty acid concentrations in ants and termites [68,69,70]; Table S3: Amino acid concentrations across ants and termites (g/kg) [25,44,68,71,72]; Table S4: Vitamin concentrations across ant and termite species [47,48,73,74,75]; Table S5: Mineral concentrations across ant and termite species [25,44,47,68,71,73,74].
