# Supplementary material for: To Save Pangolins: A Nutritional Perspective
Source: Animals (Basel). 2022 Nov 14;12(22):3137. doi: 10.3390/ani12223137 (PMC9686612; doi:10.3390/ani12223137)
Supplement: Supplementary file 1 [file animals-12-03137-s001.zip › animals-2001151-supplementary.pdf]

Supplementary Materials:

Table S1. Macronutrient concentrations across ant and termite species (g/100g).

Species	Crude protein	Crude fat	Total sugar	Moisture	Crude ash	References
Tetramorium bicarinatum	56.91	25.44	8.42	/	/	[22]
Camponotus herculeanus	61.03	8.25	15.78	/	/	[22]
Coptotermes formosanus	50.62	41.60	0.48	/	/	[22]
Odontotermes formosanus	50.62	41.60	0.42	/	/	[8]
Myrmica rubra	56.81	25.45	7.90	/	/	[8]
Polyrhachis dives	56.15	25.10	7.20	/	/	[8]
Odontotermes formosanus (workers)	63.02	5.14	2.31	/	/	[67]
The *Odontotermes formosanus* have winged breeding ants	34.69	57.00	1.20	/	/	[67]
Young *Odontotermes formosanus* and beds	23.84	2.54	3.12	/	/	[67]
Odontotermes formosanus' beds	11.50	0.71	2.50	/	/	[67]
Polyrhachis vicina Roger	57.60	20.20	/	7.10	11.10	[23]
Black ants in Changbai mountain region	52.10	17.20	/	8.70	17.00	[23]
Polyrhachis vicina Roger	57.60	20.20	/	7.10	11.10	[23]
Polyrhachis vicina Roger	54.51	11.29	2.78	10.58	5.72	[23]
Polyrhachis vicina Roger	62.40	22.60	2.90	8.60	3.50	[24]
Formica truncicola Forel	56.60	29.10	3.50	7.30	3.50	[24]
Coptotermes formosanus Shiraki	60.00	36.00	/	/	4	[30]
Polyrhachis vicina Roger	58.83	21.37	4.99	6.25	5.16	[25]
Oecophylla smaragdina	57.89	16.62	10.46	8.94	4.19	[25]
Macrotermes denticulatus	49.92	14.21	9.89	11.20	3.60	[25]

/ The data was not measured.

Table S2. Fatty acid concentrations in ants and termites.

Species	Stearic acid	Oleic acid	Linoleic acid	Linolenic acid	Palmitic acid	Palmitoleic acid	Myristic acid	Arachidic acid	Pentadecanoic acid	References
	C18:0	C18:1	C18:2	C18:3	C16:0	C16:1	C14:0	C20:4	C15:0	
Odontotermes formosanus	7.43	34.47	32.04	0.06	11.59	10.31	/	<0.01	0.26	[68]
Formica rufa L·	3.84	66.21	1.93	1.20	21.02	10.90	0.67	/	/	[69]
Polyrhachis vicina Roger	2.72	43.21	2.03	1.91	19.32	14.32	0.56	/	/	[69]
Macrotermes annandalei	9.98	51.14	13.01	0.65	18.54	2.85	0.86	0.37	0.42	[70]

%: the sum of all measured fatty acids; / The data was not measured.

Table S3. Amino acid concentrations across ants and termites (g/kg).

Species	Asp	Ser	Pro	Ala	Met	Leu	Phe	Lys	Cys	Thr	Glu	Gly	Val	Ile	Tyr	His	Arg	Trp	References
Polyrhachis vicina Roger	30	33	29	36	6	26	13	18	/*	20	52	51	29	19	27	19	18	13	[71]
Macrotermes annandalei	51	27	32	52	8	39	23	34	6	27	75	29	33	45	34	17	38	/	[72]
Macrotermes annandalei' beds	14	8	7	7	1	8	6	5	1	7	14	6	8	6	1	2	6	/	[72]
Polyrhachis vicina Roger	37	16	28	48	19	23	15	27	3	10	49	45	34	18	19	13	19	11	[25]
Oecophylla smaragdina	41	11	27	37	18	22	18	18	4	12	51	52	35	14	13	12	13	10	[25]
Macrotermes denticulatus	35	14	24	44	14	34	18	24	4	12	56	37	27	19	18	13	11	9	[25]
Yunnan yellow ant	32	20	43	37	14	32	12	20	2	17	46	32	23	23	15	9	18	/	[44]
Odontotermes formosanus	29	16	17	23	10	22	16	24	/*	17	43	18	22	14	24	11	18	2	[68]

/ The data was not measured; /* The data value is not shown because it is too low.

Table S4. Vitamin concentrations across ant and termite species.

Species	Vit. E	Vit. B_1	Vit. B_2	Vit. B_{12}	Vit. A	Vit. C	Vit. B_6	folic acid	Vit. D	References
	IU/kg	mg/kg	mg/kg	ug/kg	IU/kg	IU/kg	mg/kg	mg/kg	IU/kg	
Formica sanguinea Latr	211	3.3	26.5	A little	/	/	/	/	/	[73]
Polyrhachis vicina Roger	123	2.6	20.1	A little	/	/	/	/	/	[73]
Polyrhachis vicina Roger	23	1.3	5.2	/	28333.3	48	1.4	0.6	/	[48]
Polyrhachis vicina Roger	20	1.0	4.1	/	32666.7	22	1.8	0.5	/	[75]
Formica cunicularia Latreille	156	12.5	8.8	/	80333.3	/	/	/	8000	[74]
Polyrhachis vicina Roger	131	14.3	9.8	/	87333.3	/	/	/	8000	[74]
Polyrhachis vicina Roger	24	1.5	5.0	/	28666.7	44	1.6	5.8	/	[47]

/ The data was not measured.

Table S5. Mineral concentrations across ant and termite species.

Species	Ca	P	Mg	Na	K	Fe	Zn	Cu	Mn	Se	References
	g/kg	g/kg	g/kg	g/kg	g/kg	mg/kg	mg/kg	mg/kg	mg/kg	mg/kg	
Odontotermes formosanus	/	/	2.00	/	/	2200	180	/	1000	0.38	[68]
Formica sanguinea Later	1.89	4.26	1.42	6.64	7.58	617	232	15	182	/	[73]
Polyrhachis vicina Roger	2.69	2.02	0.96	1.26	5.08	1050	111	33	160	/	[73]
Polyrhachis vicina Roger	1.30	/	1.05	1.04	5.37	2565	140	27	306	0.44	[71]
Formica cunicularia Latreille	2.32	4.00	1.52	/	9.54	180	119	21	388	0.15	[74]
Polyrhachis vicina Roger	1.56	4.15	0.87	/	5.27	234	96	17	248	0.20	[74]
Polyrhachis vicina Roger	2.14	5.03	1.05	1.64	11.50	1142	289	44	83	0.20	[25]
Oecophylla smaragdina	1.11	7.04	1.00	3.20	11.80	594	290	34	215	0.24	[25]
Macrotermes denticulatus	1.03	6.89	1.12	1.49	10.21	780	241	40	201	0.18	[25]
Yunnanyellow ant (from Mendine)	0.29	/	/	/	5.40	280	200	30	185	/	[44]
Yunnanyellow ant (from Yongde)	0.21	/	/	/	3.20	250	195	33	170	/	[44]
Polyrhachis vicina Roger (wild)	1.69	2.01	0.43	/	/	704	164	31	326	0.27	[47]
Polyrhachis vicina Roger (raised)	1.68	2.01	0.45	/	/	700	168	31	343	0.26	[47]

/ The data was not measured.
